# Comparison of the Ocular Microbiomes of Dry Eye Patients With and Without Autoimmune Disease

**DOI:** 10.3389/fcimb.2021.716867

**Published:** 2021-09-22

**Authors:** Yun Qi, Yong Wan, Tianhui Li, Ming Zhang, Yu Song, Yaguang Hu, Yining Sun, Li Li

**Affiliations:** ^1^Department of Ophthalmology, The First Affiliated Hospital of Xi’an Jiaotong University, Xi’an, China; ^2^Department of Geriatric Surgery, The First Affiliated Hospital of Xi’an Jiaotong University, Xi’an, China; ^3^The Key Laboratory of Biomedical Information Engineering, Ministry of Education, Department of Biomedical Engineering, School of Life Science and Technology, Xi’an Jiaotong University, Xi’an, China; ^4^Department of Rheumatology, The First Affiliated Hospital of Xi’an Jiaotong University, Xi’an, China

**Keywords:** dry eye, autoimmune disease, ocular surface microbiome, 16S rRNA gene amplicon sequencing, *Corynebacterium*, *Pelomonas*

## Abstract

**Purpose:**

The pathogenesis of dry eye concomitant with autoimmune disease is different from that of dry eye without autoimmune disease. The aim of this study was to explore differences in the microbiota diversity and composition in dry eye with and without autoimmune disease.

**Methods:**

Swab samples from the inferior fornix of the conjunctival sac were obtained from dry eye patients without autoimmune disease (*n* = 49, dry eye group) and from those with autoimmune disease (*n* = 38, immdry eye group). Isolated bacterial DNAs from swabs were analyzed with 16S rRNA amplicon sequencing.

**Results:**

Analysis of the alpha diversity revealed no significant differences between subjects in the dry eye and immdry eye groups. Those in the immdry eye group had a distinct microbial composition compared with those in the dry eye group. The combination of the genera *Corynebacterium* and *Pelomonas* distinguished subjects in the immdry eye group from those in the dry eye group, with an area under the curve of 0.73 (95% CI = 0.62–0.84). For the same bacteria, the correlations between microbe abundance and the ocular surface parameters were different in the two groups. In addition, the functions of the microbial communities were altered in the two groups.

**Conclusions:**

Our study demonstrates changes in the composition and function of the ocular microbiome between subjects in the immdry eye and dry eye groups, which suggests that the potential pathogenesis is different.

## Introduction

Dry eye is the most frequent ocular feature of autoimmune diseases such as rheumatoid arthritis (RA), systemic lupus erythematosus (SLE), systemic sclerosis, and idiopathic Sjögren syndrome (SS). The proportions of dry eye in these autoimmune diseases are 71.4% (Abd-Allah et al., 2020), 36% (Wangkaew et al., 2006), 64.7% (Szucs et al., 2019), and 35% (Jacobsson et al., 1989), respectively. Thus, autoimmune disease-associated dry eye is one of the most important types of dry eye. Compared with that of dry eye patients without autoimmune disease, cell injury on the ocular surface is more serious and treatment is more difficult for dry eye subjects with autoimmune disease (Guannan et al., 2018). However, the specific mechanism of this difference is not clear.

A large number of microorganisms living on the surface of mammals have a highly co-evolutionary relationship with the autoimmune system. The mammalian immune system plays an important role in maintaining the balance of resident microbial communities. At the same time, resident bacteria profoundly shape mammalian immunity (Hooper et al., 2012). More and more evidence shows that the gut microbiome plays a key role in the pathogenesis of autoimmune diseases such as diabetes, rheumatoid arthritis, and inflammatory bowel disease (Scher and Abramson, 2011; Halfvarson et al., 2017; Vatanen et al., 2018). However, whether autoimmune diseases affect the ocular microbiome of dry eye is not clear.

There is increasing evidence that the microbiome plays a key role in ocular health and diseases. Changes of the ocular microbiome in patients with dry eye and in healthy subjects have been reported (De Paiva et al., 2016; Li et al., 2019; Kittipibul et al., 2020). Alterations of the ocular microbiome have been hypothesized to contribute to the pathophysiology of dry eye. However, these studies did not group the dry eye disease according to the autoimmune background. Herein, we compared the ocular surface microbiome between dry eye patients with autoimmune disease and those without autoimmune disease using 16S ribosomal RNA (rRNA) sequencing. We also examined the relationship between microbes and the ocular surface parameters. Finally, we analyzed the functional and metabolic differences in the microbial communities between the two groups.

## Material and Methods

### Subject Recruitment

This study adhered to the tenets of the Declaration of Helsinki and was approved by the Medical Ethics Committee of The First Affiliated Hospital of Xi’an Jiaotong University. All participants completed an informed consent form. All samples were collected from January to May 2020. The study was conducted on 49 dry eye patients without autoimmune disease (dry eye group) and 38 dry eye patients with autoimmune disease (immdry eye group). Patients were recruited from the outpatient department of the Department of Ophthalmology, the First Affiliated Hospital of Xi’an Jiaotong University.

Dry eye diagnoses were established according to the Tear Film and Ocular Surface Society’s Dry Eye Workshop (TFOS DEWS) II criteria (Wolffsohn et al., 2017). All patients were examined by an ophthalmologist using a slit lamp. The inclusion criteria were as follows: patients presenting with dry eye symptoms, such as burning, foreign body sensation, itching dryness, and photophobia; ocular surface disease index (OSDI) ≥13; and Schirmer’s test (without local anesthesia) ≤5 mm. The exclusion criteria include: use of topical antibiotics in the previous 4 weeks, use of contact lenses in the previous 4 weeks, history of ocular and periocular infection in the previous 4 weeks, and eye surgery within 3 months. All enrolled patients were at least 18 years of age.

Patients’ clinical histories were documented. Those with a history of autoimmune disease and are seropositive (autoimmune antibodies, C-reactive protein, rheumatoid factor, thyroid autoantibodies) were classified as the dry eye with autoimmune disease group (immdry eye; **Table 1**). Patients without a history of autoimmune disease had a blood test to check for blood routine parameters and thyroid function and the presence of C-reactive protein, rheumatoid factor, and autoimmune antibodies. Those whose results were within the normal range were included in the dry eye group (dry eye; **Table 1**).

**Table 1:** Characteristics of the two groups.

	Dry eye group	Immdry eye group	
Description	Dry eye patients without autoimmune disease	Dry eye patients with autoimmune disease	
Samples	*n* = 49	*n* = 38	
Autoimmune disease	None	SLE	*n* = 10
		RA	*n* = 13
		SS	*n* = 6
		Systemic sclerosis	*n* = 5
		Graves’ disease	*n* = 4

SLE, systemic lupus erythematosus; RA, rheumatoid arthritis; SS, Sjögren syndrome.

### Sample Collection

Conjunctival swabs were performed in 49 eyes of 49 dry eye patients and in 38 eyes of 38 immdry eye subjects. Three minutes after instilling topical anesthesia (0.4% benoxir solution; Santen, Osaka, Japan), a sterile cotton swab was applied three times from the medial to the lateral side of the inferior fornix of the conjunctival sac without touching the eyelids. The swabs were then placed in sterile transport media. The samples were conserved in lysis solution and stored in a refrigerator (at −80°C) until use.

### DNA Extraction, PCR Amplification, and 16S rRNA Gene Amplicon Sequencing

DNA was extracted using the magnetic bead extraction method with a customized BGI kit (Beijing Genomics Institute, Guangdong, China). The concentration of bacterial DNA was measured using a NanoDrop 2000 ultramicro-spectrophotometer (Thermo Scientific, Waltham, MA, USA). The V3–V4 region of the 16S rRNA gene was amplified from 30 ng of qualified genomic DNA samples and the corresponding fusion primers (341F: 5′-ACTCCTACGGGAGGCAGCAG-3′ and 806R: 5′-GGACTACHVGGGTWTCTAAT-3′) used to configure the PCR reaction system and to perform PCR amplification. The PCR amplification products were purified with Agencourt AMPure XP magnetic beads (Fisher Scientific, Hampton, NH, USA), dissolved in elution buffer, and then labeled. The Agilent 2100 Bioanalyzer (Agilent, Santa Clara, CA, USA) was used to detect the fragment range and the concentration of the library. Qualified libraries were selected for sequencing on the HiSeq platform based on the size of the inserted fragments.

### Bioinformatics Analysis of 16S rRNA Gene Amplicon Sequencing

The Quantitative Insights Into Microbial Ecology software (QIIME2 v.1.9.1) was used to analyze the 16S rRNA sequencing data. To filter the sequencing reads and construct a feature table, the DADA2 software was used, wrapped in QIIME2, with no truncation in both the forward and reverse directions. Spliced tags were clustered into operational taxonomic units (OTUs) using the USEARCH software (v.7.0.1090). Tags with a similarity of more than 97% are clustered into an OTU. The venndiagram package of R (v.3.1.1) was used to make a Venn diagram showing the number of common and unique OTUs between the two groups. Values for the alpha diversity (ACE index, Chao index, *S*_obs_, Shannon’s index, and Simpson index), beta diversity, and principal coordinate analysis (PCoA) based on the unweighted and weighted UniFrac metrics were generated by QIIME v.1.9.1. Linear discriminant analysis effect size (LEfSe) was used to determine the features that most likely explain the differences between the groups. The metagenomes of the ocular microbiome were imputed from the 16S rRNA sequences with PICRUSt2 v.2.3.0-b (Phylogenetic Investigation of Communities by Reconstruction of Unobserved States). In addition, PICRUSt2 was utilized to predict the functional potential of the microbial community *via* marker gene sequencing profiles. Default gene bank of the Kyoto Encyclopedia of Genes and Genomes (KEGG) was used to support functional gene profiling.

### Ocular Surface Parameter Assessment

To obtain the ocular surface parameters related to tear film function, Keratograph 3 (Oculus, Wetzlar, Germany) was used to scan the tear film of all the participants. The six ocular surface parameters obtained were as follows: tear meniscus height (TMH), the first break of tear breakup time (F-BUT), average tear breakup time (A-BUT), ocular redness index (ORI), Meibomian gland dropout score (MGDS), and the lipid layer score (LLS). TMH was measured at the pupil center perpendicular to the palpebral margin. The ORI was analyzed with the R-scan software. The MGDS of the upper lid was scored using a four-grade scoring system (1–4) based on the grading scale of Arita et al. (2010). Briefly, according to the lost area of the upper eyelid Meibomian gland, we divided the MGDS into four grades: grade 1, no area loss; grade 2, lost area less than 33%; grade 3, lost area within 33%–67%; and grade 4, lost area exceeding 67%. The LLS was determined using a four-grade scoring system according to the instructions (based on the tear film scanning results). In brief, grade 1 is for rich in lipids, grade 2 for lipid balance, grade 3 for lipid deficiency, and grade 4 for severe lipid deficiency.

### Statistical Analysis

We analyzed differences in the ocular surface parameters between the two groups with the Wilcoxon rank-sum test. The same test was used to analyze the alpha diversity metrics and beta diversity. We applied LEfSe analysis to identify differences in taxa between the dry eye and immdry eye patients. This method first uses the non-parametric factorial Wilcoxon rank-sum test to detect features with significant differential abundance and then uses linear discriminant analysis (LDA) to calculate the effect size of each feature. Statistical analyses were performed with R software (v.3.4.1). Diagnostic performance was estimated using the relative operating characteristic (ROC) curve. We then calculated the area under the curve (AUC) with the pROC package. Other statistical analyses included a chi-squared test and partial Spearman’s rank correlation (PResiduals package). *P*-values <0.05 were considered statistically significant.

## Results

### Clinical Characteristics

A total of 87 swabs were collected from 49 dry eye patients and 38 immdry eye patients. Age and gender were matched between the two groups. All patients were examined with the Keratograph to evaluate the F-BUT, A-BUT, TMH, ORI, MGDS, and LLS. **Table 2** presents the detailed demographic and clinical features of the cohorts.

**Table 2:** Ocular surface parameter assessment of the groups.

	Dry eye group	Immdry eye group	*p*-value
Age (years), median (min–max)	42 (21–69)	50 (23–65)	0.051
Sex, female, *n* (%)	39 (79.6%)	32 (84.2%)	0.78
TMH	0.15 ± 0.03	0.14 ± 0.09	0.08
F-BUT	5.59 ± 3.79	4.28 ± 2.00	0.33
A-BUT	7.72 ± 4.85	5.89 ± 3.12	0.15
ORI	1.17 ± 0.42	1.28 ± 0.44	0.12
MGDS, *n* (%)			0.03
G-1	1 (2.0)	0 (0)	
G-2	39 (79.6)	23 (60.5)	
G-3	6 (12.2)	12 (31.6)	
G-4	3 (6.1)	3 (7.9)	
LLS, *n* (%)			0.63
G-1	0 (2.0)	2 (5.3)	
G-2	23 (30.6)	10 (26.3)	
G-3	12 (65.3)	23 (60.5)	
G-4	3 (6.1)	3 (7.9)	

TMH, tear meniscus height; F-BUT, first break of tear breakup time; A-BUT, average tear breakup time; ORI, ocular redness index; MGDS, Meibomian gland dropout score; LLS, lipid layer score.

### Immdry Eye Subjects Harbored an Altered Bacterial Eye Microbiome

**Figure 1:**
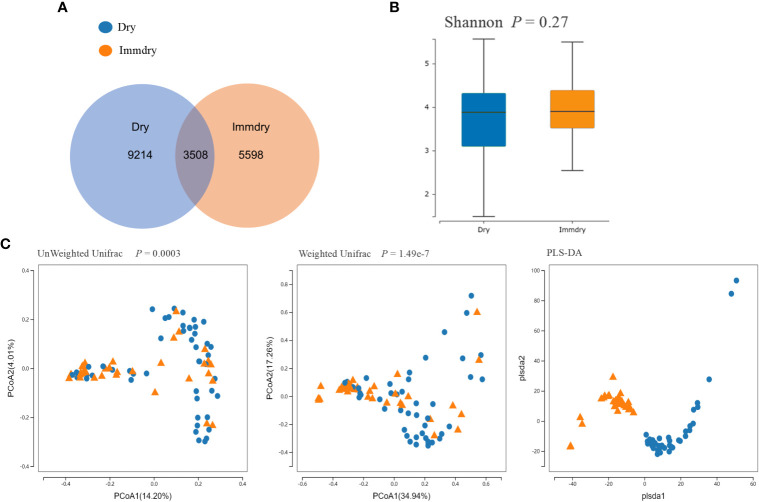
Comparisons of the alpha diversity and beta diversity between dry eye and immdry eye patients. **(A)** Venn diagram showing overlaps of the operational taxonomic units (OTUs) between the two groups. **(B)** Alpha diversity of the dry eye microbiome similar to that of immdry eye, as quantified by diversity indices (Shannon, *p* = 0.27). **(C)** Principal coordinate analysis (PCoA) of bacterial beta diversity based on the unweighted (*p* = 0.0003) and weighted (*p* = 1.49e−7) UniFrac distances and partial least squares–discriminant analysis (PLS-DA) dissimilarity. Dry eye and immdry eye subjects are indicated in *blue* and *orange*, respectively.

Analysis of the alpha diversity revealed no significant differences between the dry eye and immdry eye subjects (three richness indices: Ace, Chao, and *S*_obs_, both *p* = 0.14; diversity indices: Shannon and Simpson, *p* = 0.27 and *p* = 0.06, respectively; coverage of the sample library, *p* = 0.94) (**Figure 1B**). However, analysis of the beta diversity calculated with the unweighted (*p* = 0.0003) and weighted UniFrac (*p* = 1.49e−7) and PLS-DA (partial least squares–discriminant analysis) distances revealed that the bacterial microbiome composition of the immdry eyes patients was different from that of dry eye subjects (**Figure 1C**).

### Bacteria Differentially Abundant in the Dry Eye and Immdry Eye Groups

**Figure 2:**
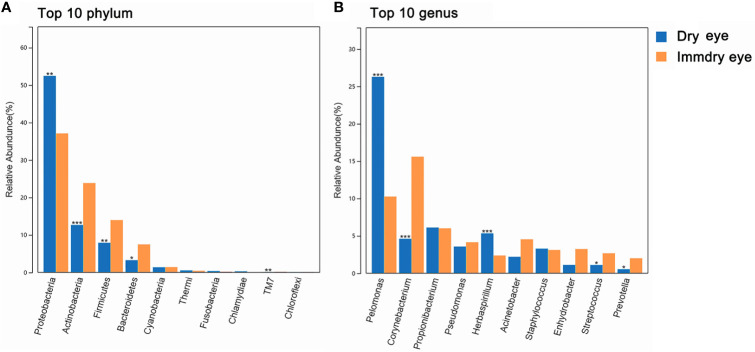
Comparisons of the taxonomic classifications between the dry eye group and the immdry eye group. **(A)** Top 10 phyla in the two groups. **(B)** Top 10 genera in the two groups. Dry eye and immdry eye subjects are indicated in *blue* and *orange*, respectively. *p < 0.05, **p < 0.01, ***p < 0.001.

For the two groups at the phylum level (**Figure 2A**), the top 10 phyla were *Proteobacteria*, *Actinobacteria*, *Firmicutes*, *Bacteroidetes*, *Cyanobacteria*, *Thermi*, *Fusobacteria*, *Chlamydiae*, *TM7*, and Chloroflexi. Proteobacteria were markedly higher in the dry eye group compared with the immdry eye group (52% *vs*. 37%, *p* = 0.002). *Actinobacteria*, *Firmicutes*, *Bacteroidetes*, and *TM7* were more abundant in the immdry eye group compared with the dry eye group (23% *vs*. 13%, *p* = 0.001; 14% *vs*. 8%, *p* = 0.006; 7% *vs*. 3%, *p* = 0.011; and 0.18% *vs*. 0.08%, *p* = 0.004, respectively).

For the two groups at the genus level (**Figure 2B**), the top 10 genera were *Pelomonas*, *Corynebacterium*, *Propionibacterium*, *Pseudomonas*, *Herbaspirillum*, *Acinetobacter*, *Staphylococcus*, *Enhydrobacter*, *Streptococcus*, and *Prevotella*. *Pelomonas* and *Herbaspirillum* were markedly higher in the dry eye group compared with the immdry eye group (26% *vs*. 10%, p = 0.0002; 2% *vs*. 5%, p = 0.0005, respectively). *Corynebacterium*, *Streptococcus*, and *Prevotella* were more abundant in the immdry eye group compared with the dry eye group (16% *vs*. 5%, p = 0.0001; 3% *vs*. 1%, p = 0.04; and 2% *vs*. 1%, p = 0.04, respectively).

### Bacterial Biomarkers in the Dry Eye and Immdry Eye Groups

**Figure 3:**
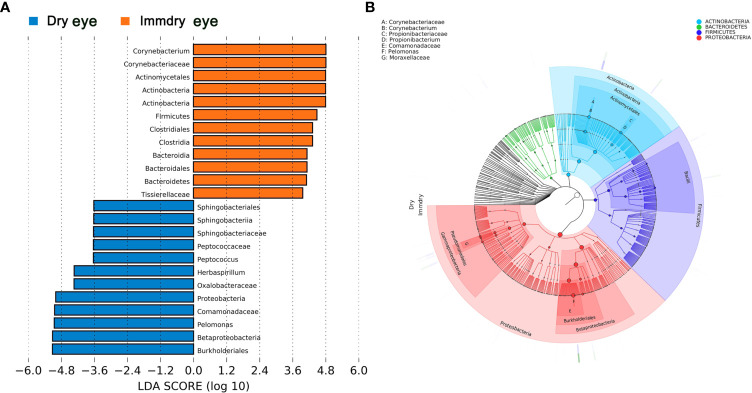
Bacterial biomarkers identified with the linear discriminant analysis effect size (LEfSe) algorithm. **(A)** Linear discriminant analysis (LDA) scores with the LEfSe tool for taxa, with LDA scores >3.6 and *p* < 0.05 shown in the histogram. **(B)** Cladogram displaying the relations between taxa at different taxonomic levels. Each *circle* represents a hierarchy, followed by phylum, class, order, family, and genus. Different phyla are marked with *different colors*. The *size of the nodes* represents the taxon abundance.

**Figure 4:**
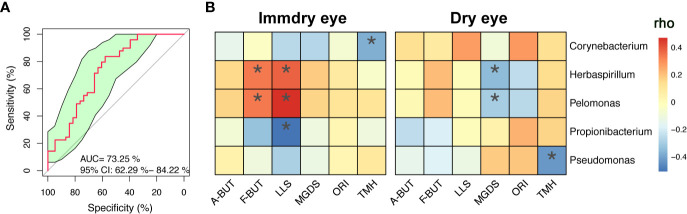
Correlations between microbe abundance and the ocular surface parameters in the two groups. **(A)** Classification performance of the multivariable logistic regression model using relative abundance of genera (combination of *Corynebacterium* and *Pelomonas*) was assessed using area under the ROC. **(B)** Heatmap showing partial Spearman’s correlation coefficients between the five genera and the ocular surface parameters in the immdry eye and dry eye groups. *A-BUT*, average break of tear breakup time; *F-BUT*, the first break of tear breakup time; *LLS*, lipid layer score; *MGDS*, Meibomian gland dropout score; *ORI*, ocular redness index; *TMH*, tear meniscus height; *ROC*, relative operating characteristic. *p < 0.05.

We further analyzed the bacterial community structure associated with dry eye and immdry eye using LEfSe, an algorithm for high-dimensional biomarker discovery that uses LDA to estimate the effect size of each taxon that is differentially represented in two groups (**Figure 3**). The phyla biomarkers were *Actinobacteria*, *Firmicutes*, and *Bacteroidetes* for the immdry eye group and *Proteobacteria* for the dry eye group. At the genus level, the identified biomarkers were *Corynebacterium* for the immdry eye group and *Pelomonas* for the dry eye group (**Figure 3A**). We next assessed the potential value of using *Corynebacterium* and *Pelomonas* as biomarkers. We found that the combination of *Corynebacterium* and *Pelomonas* could discriminate immdry eye from dry eye with an AUC of 0.73 (95% CI = 0.62–0.84) (**Figure 4A**).

### Correlation Between Microbe Abundance and Ocular Surface Parameters in the Dry Eye and Immdry Eye Groups

To investigate the correlation between microbe abundance and the ocular surface parameters in the two groups, we chose the top five bacteria genera (**Figure 2B**) and performed Spearman’s rank-based correlation test, taking age and gender into account. As shown in **Figure 4B**, the abundance of *Corynebacterium* was negatively correlated with TMH in the immdry eye group. The abundance of *Herbaspirillum* and *Pelomonas* was positively correlated with F-BUT and LLS in the immdry eye group, while it was negatively correlated with MGDS in the dry eye group. The abundance of *Propionibacterium* was negatively correlated with LLS in the immdry eye group. The abundance of *Pseudomonas* was negatively correlated with TMH in the dry eye group. The results for the same bacteria showed that the correlations between microbe abundance and the ocular surface parameters were different in the two groups. Therefore, we speculated that the same bacteria may have played different roles in the pathogenesis of dry eye in these different groups.

### Microbial Functional Alteration

**Figure 5:**
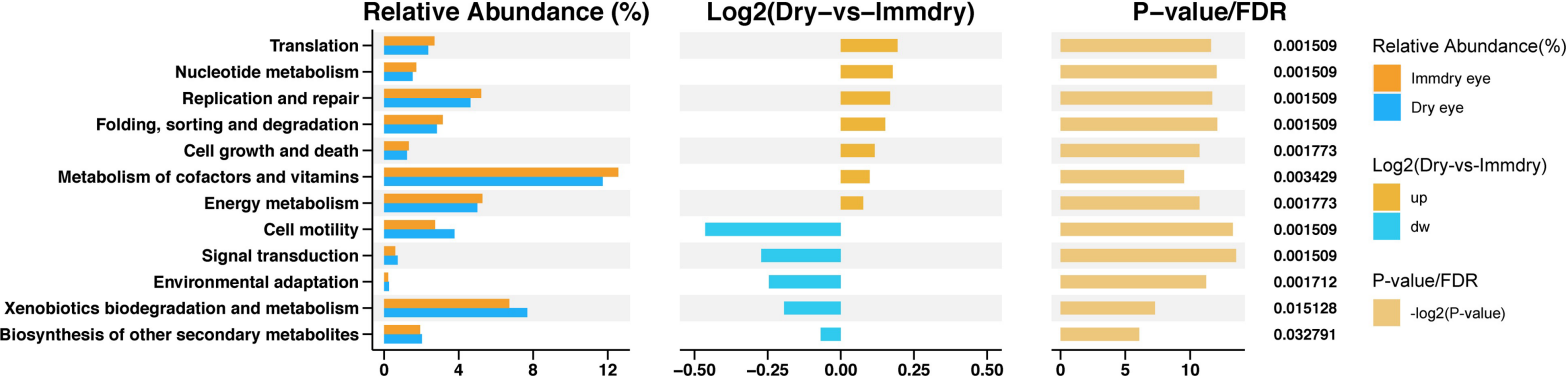
Functional analysis of the predicted metagenomes. On the *left* is the relative abundance histogram for each group, in the *middle* is the log2 value of the mean relative abundance ratio of the same pathway in the two groups, and on the *right* is the −log2(*p*-value) and false discover rate (FDR) value obtained using the Wilcoxon test. If the *p*-value and FDR were less than 0.05, then there is a significant difference between the two groups.

To study the functional alterations of the microbial communities in the dry eye and immdry eye groups, we next inferred the metagenomes from the 16S rRNA data and analyzed the functional potential of the microbiome using PICRUSt2 (**Figure 5**). The results showed 12 KEGG categories with significant differential abundance between the two groups. We found that pathways involved in translation, nucleotide metabolism, replication and repair, folding, sorting and degradation, cell growth and death, metabolism of cofactors and vitamins, and energy metabolism were increased in the immdry eye group. However, cell motility, signal transduction, environmental adaptation, xenobiotics biodegradation and metabolism, and other secondary metabolites were decreased in the immdry eye group.

## Discussion

There is growing evidence that alterations in the ocular microbiome are associated with dry eye (Li et al., 2019; Willis et al., 2020). However, it is not clear whether there is a difference in the composition of the ocular microbiome between dry eye with autoimmune disease and that without. Herein, we delineated the community structure of the ocular microbiome between the dry eye and immdry eye groups. It was demonstrated that the immdry eye group had significant alterations of beta diversity compared to the dry eye group. According to the microbial characteristics, we built a model that can distinguish subjects in the immdry eye group from those in the dry eye group. Furthermore, we compared the correlations between the dominant bacterial abundance and the ocular surface parameters of the two groups. Finally, we analyzed the functional and metabolic changes in the microbial communities between the dry eye and immdry eye groups.

The ocular microbe beta diversity exhibited significant differences between the immdry eye group and dry eye group. To the best of our knowledge, no study has compared the differences of the ocular microbiome between two groups of patients. Many studies have indicated that there is a reduction in the intestinal microbial diversity in many autoimmune diseases. For example, a reduction in the intestinal microbiome diversity has been discovered in Crohn’s disease, type 1 diabetes mellitus, allergies, and multiple sclerosis (Mosca et al., 2016). The mammalian immune system plays an essential role in maintaining homeostasis with resident microbial communities (Hooper et al., 2012). It controls the microbiome composition (Kau et al., 2011). Several immune effectors (the mucus layer produced by goblet cells, epithelial antibacterial proteins, and immunoglobulin A secreted by lamina propria plasma cells) and immune cells function together to stratify luminal microbes and minimize bacterial penetrations into the body (Hooper et al., 2012). Therefore, defects in the host immune system can affect these microbial communities. The mechanism might be the result of the interactions between the ocular surface immune system and the microbiome.

We defined the community structure of the ocular microbiome in immdry eye patients using high-throughput 16S rRNA gene sequencing. We found alterations at the phylum level with increases in *Actinobacteria*, *Firmicutes*, *Bacteroidetes*, and *TM7* and a reduction in *Proteobacteria*. At the genus level, we observed microbiome increases of *Corynebacterium*, *Streptococcus*, and *Prevotella* and reductions of *Pelomonas* and *Herbaspirillum*. The microbiomes of the phyla *Proteobacteria*, *Actinobacteria*, *Firmicutes*, and *Bacteroidetes* were the most abundant in the dry eye group. This result is consistent with other studies (Watane et al., 2019; Andersson et al., 2021). However, at the genus level, the main bacteria in our study were different from those reported in other studies (Watane et al., 2019; Andersson et al., 2021). This result can be explained by our classification of the dry eye patients in this study into two groups, with or without autoimmune diseases; there was no such division in other studies. *Corynebacterium* is one of the normal bacterial residents of the skin and mucosal surfaces, including the nose and ocular surfaces (Hoshi et al., 2020). In our study, *Corynebacterium* was increased in the immdry eye compared with the dry eye group. This genus has been shown to be associated with autoimmunity. Because mycolic acids and the cell wall architecture of *Corynebacterium* are well-known effective immune-stimulatory compounds, they can affect macrophage function (Burkovski, 2018). Thus, *Corynebacterium* may be driving the immune changes in dry eye disease.

The most interesting result in our study was the correlations between microbe abundance and the ocular surface parameters in the two groups. The same bacteria were analyzed in both groups, but the correlations between microbe abundance and the ocular surface parameters were different in the two groups. This can be explained by the following: 1) choosing the dominant flora according to the top five bacteria genera led to there being a significant difference in the bacterial abundance between the two groups. Rivett et al. found that abundance determines the functional role of bacterial phylotypes in complex communities (Rivett and Bell, 2018). Therefore, we speculated that the change in flora abundance may have caused a change in the ocular surface parameters. 2) There were different immune states in the two groups, and the function of the same bacteria may be altered in different immune states.

The human microbiome is an extremely complex ecosystem considering the number of bacterial species, their interactions, and its variability over space and time (Cho and Blaser, 2012). The field of microbiome science is still in its infancy. At present, most studies have focused on the impact of intestinal flora changes on systemic diseases; research on ocular surface flora has been less. Due to the diversity and complexity of flora, the functional roles of bacterial taxa within communities are difficult to study (Rivett and Bell, 2018). The mainstream approach uses metagenomic or meta-transcriptomic data to infer functions. We propose that the ocular surface is a good model for studying the function of flora in humans: 1) the parameters of ocular surface function are easy to quantify and observe; 2) specimens on the ocular surface are easy to collect; and 3) the ocular surface is sensitive because there are many nerves distributed on the ocular surface compared to the intestinal cavity, which also means that the conjunctival sac receives less interference factors than the intestinal cavity.

In the study of Guannan et al., the morbidity of dry eye in normal populations was 28.3%, and for patients with systemic autoimmune disease, this was 51.7% (Guannan et al., 2018). The authors found that the condition of dry eye in patients with systemic autoimmune diseases was more serious and that the percentage of conjunctival epithelial cell apoptosis was higher than that in the control group. The specific mechanism is not clear. We studied the functional and metabolic changes of the microbial communities in the two groups. Of note is that cell growth and death were increased in the immdry eye group (**Figure 5**). Thus, we speculated that flora may have played a significant role in regulating epithelial cell growth and death in the immdry eye group.

The major advantage of our study was that the ocular surface parameters of dry eye were obtained for further analysis. Our research has the following limitations, however. Firstly, the gene sequencing method we used is 16S rRNA. Compared with metagenome sequencing, 16S rRNA gene sequencing is inferior in the species level and in function analysis. Secondly, our study may be affected by confounding factors such as systemic drug use, the severity of the immune diseases, and the environment. Thirdly, ours is a single-center research, and it is unclear whether our results can be verified in other centers.

Nonetheless, our results supported a comprehensive investigation of ocular surface microbiomes between the dry eye and immdry eye groups and provided novel insights into the pathogenesis of dry eye disease.

## Data Availability Statement

The data presented in the study are deposited in the NCBI BioProject repository, accession number PRJNA743593.

## Ethics Statement

The studies involving human participants were reviewed and approved by the Medical Ethics Committee of The First Affiliated Hospital of Xi’an Jiaotong University. The patients/participants provided their written informed consent to participate in this study.

## Author Contributions

YQ participated in the data analysis and writing of the paper. YW and TL participated in the data analysis. MZ, YuS, and YH participated in data collection and revising the paper. YiS participated in the research design. LL designed the study and wrote and revised the paper. All authors contributed to the article and approved the submitted version.

## Funding

This work was supported by the National Natural Science Foundation of China (no. 81800824), the Natural Science Basic Research Plan in Shaanxi Province of China (no. 2019JQ-956), the Key Research and Development Plan of Shaanxi Province (no. 2020ZDLSF02-06), and the Clinical Research Award of the First Affiliated Hospital of Xi’an Jiaotong University (no. XJTU1AF-CRF-2018-014).

## Conflict of Interest

The authors declare that the research was conducted in the absence of any commercial or financial relationships that could be construed as a potential conflict of interest.

## Publisher’s Note

All claims expressed in this article are solely those of the authors and do not necessarily represent those of their affiliated organizations, or those of the publisher, the editors and the reviewers. Any product that may be evaluated in this article, or claim that may be made by its manufacturer, is not guaranteed or endorsed by the publisher.
